# A Global Analysis of the Distribution Patterns of Style‐Length Polymorphisms Across Angiosperms

**DOI:** 10.1002/ece3.72659

**Published:** 2026-04-20

**Authors:** Letícia Rodrigues Novaes, Violeta Simón‐Porcar, Rafael Molina‐Venegas, Juan Arroyo

**Affiliations:** ^1^ Department of Plant Biology and Ecology University of Seville Seville Spain; ^2^ Estación Biológica de Doñana (EBD‐CSIC) Seville Spain

**Keywords:** biogeography, hermaphroditic plants, heterostyly, mating system, outcrossing, pollination

## Abstract

Unraveling the evolutionary and biogeographical factors that drive the widespread occurrence of outcrossing mechanisms in plants is key to understanding their prevalence. Style‐length polymorphisms (SLP) are labile outcrossing traits that have evolved repeatedly in several angiosperm lineages sharing moderately specialized pollination features, reflecting a convergent strategy to optimize outcrossing efficiency. Notably, over 50% of known genera exhibiting SLP belong to the Rubiaceae family, which, beyond harboring floral architectures that promote pollination precision, is characterized by a predominantly tropical distribution and high species diversity. This fact suggests that the evolution and distribution of SLP could be associated with specialized pollinator interactions prevalent in tropical regions (tropicality‐driven hypothesis) and with high species richness, potentially boosting the chance of random appearance of SLP (diversity‐driven hypothesis). We examined the biogeographical correlates of SLP across all angiosperm genera on a global scale to test the tropicality‐ and diversity‐driven hypotheses. We conducted phylogenetic logistic regression models to assess the prevalence of SLP genera in the tropics and biodiversity hotspots (areas of exceptional species richness), accounting for phylogenetic conservatism, genus‐level species richness, and evaluating the role of the Rubiaceae family. Our analyses found no support for either the tropicality‐ or the diversity‐driven hypotheses once phylogenetic relatedness was accounted for. Despite the occurrence of SLP being significantly associated with tropical habitats, this pattern relied on the Rubiaceae family. No positive association emerged between SLP and biodiversity hotspots. Instead, phylogenetic conservatism predominantly shaped SLP distribution. Also, there was a clear positive relationship between genus‐level species richness and SLP. Our findings challenge tropicality‐ and diversity‐driven hypotheses, suggesting that SLP distribution is primarily shaped by conserved evolution. Future studies should explore further the role of SLP in species richness by explicitly assessing its potential influence on diversification patterns.

## Graphical abstract

This study tested the tropicality‐ and diversity‐driven hypotheses to explain the global distribution of style‐length polymorphisms (SLP) in angiosperms. Phylogenetic logistic regression revealed that although SLP occurrence was associated with tropical regions, this pattern was driven by the predominantly tropical family Rubiaceae and disappeared once phylogenetic relationships were accounted for. We found no support for either the tropicality‐ or diversity‐driven hypotheses; instead, SLP distribution was best explained by phylogenetic conservatism. Additionally, we found a positive association between genus‐level species richness and the occurrence of SLP.

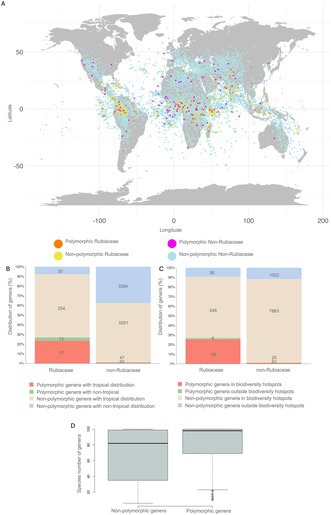

## 1 Introduction

Outcrossing is a widespread mating strategy enhancing the genetic diversity and adaptive potential of plants to environmental changes (Barrett 2015). Several mechanisms, such as dioecy, dichogamy, self‐incompatibility, or heterostyly, have evolved to promote outcrossing in different flowering plant lineages, shaped by the interaction between inherited genetic traits and ecological conditions. In a spatial context, both the evolutionary history of plant lineages and the geographic structuring of ecological factors can impact the biogeography of mating systems, particularly in animal‐pollinated plants, as has been demonstrated for various plant reproductive traits and strategies (Renner and Ricklefs 1995; Barrett et al. 2001; Moeller 2006; Ollerton et al. 2006; Hargreaves and Eckert 2014; Moeller et al. 2017; Wang, Luo, et al. 2021).

A higher prevalence of pollinator‐mediated outcrossing strategies has been reported in tropical areas (Moeller et al. 2017). In fact, plant‐pollinator interactions tend to be more specialized at lower latitudes (Olesen and Jordano 2002; Ollerton et al. 2006), and such specialization can facilitate outcrossing by ensuring precise pollen transfer between individuals, thereby minimizing self‐pollination and pollen and ovule loss (Lloyd and Webb 1992). Beyond ecological factors, the long‐term climatic stability of tropical areas has favored their high biodiversity (Pianka 1966; Hillebrand 2004; Mittelbach et al. 2007; Huntingford and Mercado 2013), including a large number of species, which in turn could also promote the repeated evolution of labile reproductive traits.

Among the most successful and studied plant outcrossing strategies are style‐length polymorphisms (SLP), including heterostyly and stigma‐height dimorphism (Darwin 1877; Ganders 1979; Lloyd and Webb 1992; Ferrero et al. 2011; Simón‐Porcar et al. 2024). These polymorphisms involve the presence of two or three floral morphs within a population, typically characterized by a reciprocal arrangement of stigma and anther heights (Barrett et al. 2000). This reciprocal placement of sex organs promotes efficient pollination between floral morphs contacting female and male sex organs with different matching parts of the pollinator's body (Darwin 1877; Ganders 1979; Keller et al. 2014), mainly in plant lineages with tubular flowers pollinated by long‐tongued insects (Simón‐Porcar et al. 2024), embodying a classic example of functionally specialized pollination interaction favoring pollination precision.

Style‐length polymorphisms have independently evolved several times in at least 34 unrelated angiosperm families but, remarkably, over 50% of SLP genera belong to the Rubiaceae (Simón‐Porcar et al. 2024). Beyond typically bearing the floral architecture associated with the evolution of SLP (Simón‐Porcar et al. 2024), this family is characterized by its predominantly tropical distribution and high species richness (Bremer and Eriksson 1992; Davis et al. 2009). These facts raise questions about the potential influence of tropicality and species richness in the occurrence of this labile outcrossing trait. Despite extensive research on the function and evolution of SLP in promoting outcrossing, most studies have focused on a particular lineage (e.g., Maguilla et al. 2021) or restricted geographic areas (e.g., Simón‐Porcar et al. 2015). As a result, the global biogeographic patterns of SLP across angiosperms remain poorly understood compared with other reproductive systems.

Here, we report the first study examining the biogeography of style‐length polymorphisms across angiosperms, testing the two following hypotheses to explain the occurrence of SLP on a global scale: (1) the tropicality‐driven hypothesis, which links the evolution of SLP to the prevalence of specialized plant‐pollinator interactions and outcrossing in tropical environments; and (2) the diversity‐driven hypothesis, which links the evolution of SLP to its chance of random appearance in highly diverse areas. To distinguish the role of both hypotheses, which are not mutually exclusive, we tested the prevalence of SLP genera in tropical regions and biodiversity hotspots, the latter being areas of exceptional species richness, which spread beyond the tropics (Myers 1988; Mittermeier et al. 2011). To take into account the evolutionary history of plant lineages, we conducted phylogenetic logistic regression using a genus‐level mega‐phylogeny of flowering plants and a data set including the geographic distribution of all style‐length monomorphic and polymorphic angiosperm genera. We evaluated the influence of phylogenetic conservatism, tropical regions, and biodiversity hotspots on the global distribution of SLP, assessing explicitly the role of the Rubiaceae family and considering the genus‐level species richness.

## 2 Materials and Methods

### 2.1 Dataset

We retrieved a genus‐level list of all known SLP taxa from the comprehensive review of Simón‐Porcar et al. (2024). Because species‐level data on the occurrence of SLP remain insufficient, and given that SLP is a labile trait that has repeatedly evolved and been lost across species and populations (e.g., Ferrero et al. 2009, 2011; Pérez‐Barrales and Arroyo 2010; Zhou et al. 2017; Barrett 2019), analyses at finer taxonomic scales would stand over inaccurate data and could therefore yield unreliable results. For this reason, we used the list compiled by Simón‐Porcar et al. (2024), which classified genera as polymorphic when at least one species exhibited SLP. The list follows strictly morphological criteria, including records of SLP linked with cryptic dioecy (eight genera) and non‐reciprocal style‐length dimorphism (five genera), which represent evolutionary phenotypic conditions close to heterostyly (Beach and Bawa 1980; Lloyd and Webb 1992). The presence of heteromorphic self‐incompatibility (HetSI) was not required to consider a genus as SLP, because although HetSI is often associated with SLP (Ganders 1979; Barrett and Cruzan 1994; Ferrero et al. 2011), it is not a necessary condition (Lloyd and Webb 1992).

We obtained a list of all known angiosperm genera from the database of Plants of the World Online (POWO 2022). This database included 13,266 genera and data on their distribution, with presence/absence records across 368 botanical countries (regions hereafter; Table S1). Each genus in the POWO list was coded as non‐polymorphic (state 0) or polymorphic (state 1), following Simón‐Porcar et al. (2024) (Table S2).

We categorized the 13,266 angiosperm genera based on their distribution in tropical regions and biodiversity hotspots (hereafter: “hotspots”). As a prior step, we classified the 368 regions. Regions were considered “tropical” if they at least partly extended into the tropics (latitudinal range ±23°27′), and “non‐tropical” otherwise. This definition follows the most conservative approach, as tropical regions have been considered in at least eight different ways (Feeley and Stroud 2018), and the chosen range encompasses the shared core across all alternatives. Although a mosaic of multiple, often unspecified, environmental variables is associated with tropicality, latitude represents a major axis of variation and serves as a reliable proxy for this condition (Wang, Ives, et al. 2021). Regions were considered to be “within hotspots” if they overlapped, at least in part, with at least one hotspot (see Mittermeier et al. 2011 for details of hotspot classification and distribution) (Table S1), and “outside” otherwise.

Genera were scored as tropical (state 1) if at least 50% of the regions in which they occurred had been scored as “tropical,” and non‐tropical (state 0) otherwise. The classification of a genus as tropical or non‐tropical using a 50% distribution threshold is arbitrary and may impact our results. To address this, a more conservative approach with a 70% threshold was employed for reclassification, resulting in similar outcomes (Tables S3–S6). Genera were scored as predominantly present within hotspots (state 1) if at least 50% of the regions in which they occurred had been scored as “within hotspots,” and absent (state 0) otherwise. The 70% threshold was applied only to tropical classification due to its gradient‐based nature, whereas hotspots are discrete and categorically defined. Because most genera in our dataset have continuous ranges, our threshold‐based coding into tropical and hotspot categories provides a reasonable approximation of their distribution patterns. The number of species for each genus was obtained using the *name_lookup* function in the R package *rgbif* (Chamberlain et al. 2025), which compiles taxonomic information from the Global Biodiversity Information Facility database.

### 2.2 Statistical Analysis

#### 2.2.1 Exploratory Analysis

As a preliminary analysis excluding phylogenetic effects, we computed chi‐square (*χ* ^2^) statistics to test, across the entire data set of angiosperm genera (*N* = 13,266), the association between (i) SLP and tropical distribution; and (ii) SLP and occurrence in hotspots (Table S3). We also used a *t*‐test to compare the number of species between SLP and non‐SLP genera. We repeated these analyses using only the genera included in the phylogenetic analyses (*N* = 9425; see below).

#### 2.2.2 Phylogenetic Analyses

We used the angiosperm phylogenetic tree from Simón‐Porcar et al. (2024). These authors derived a genus‐level time‐calibrated angiosperm phylogeny from the GBOTB tree of Smith and Brown (2018) by dropping all gymnosperm taxa and randomly selecting one species per angiosperm genera (*N* = 10,424). We used the “drop.tip” function of the R package *ape* (Paradis et al. 2004) to remove genera with missing distribution data, resulting in our final phylogenetic tree including 9425 angiosperm genera, with 206 SLP genera belonging to 32 angiosperm families (71% of all genera and 85% of SLP genera from our original database) (see Table S2 for polymorphic genera). We performed all analyses with the pruned tree (*N* = 9425).

We computed the strength of the phylogenetic signal in the occurrence of SLP using the function *phylo.d* as implemented in the R package *caper* (Orme et al. 2022). This method estimates the observed D statistic for a binary trait on a tree and compares this to the value of D found using an equal number of simulations under each of two models: a Brownian threshold model (*D* ~0, expected under phylogenetic conservatism) and a phylogenetic random model (*D* ~1, expected under phylogenetic randomness). The D values below 0 indicate very strong phylogenetic conservatism, values around 0 are consistent with Brownian motion, values close to 1 indicate randomness, and values above 1 suggest overdispersion. The function also provides *p*‐values assessing whether the observed D differs significantly from the Brownian or random expectations. To ensure that any phylogenetic effect observed for SLP is not simply driven by shared geographical distributions among related taxa, we also computed the phylogenetic signal independently for each geographical variable (occurrence in tropical regions and hotspots).

We used phylogenetic logistic regression models (Ives and Garland 2010) to test the partial influence of evolutionary relatedness, occurrence in tropical regions, and hotspots on the occurrence of SLP in the angiosperm global flora at the genus level. Beyond occurrence in hotspots, we also included the species richness of genera in the models to control its possible direct influence on the random repeated evolution of this trait. The models were performed using the *phyloglm* function in the R package *phylolm* (Ho and Ané 2014). We estimated the relative contribution of each variable with the partial $R_{\mathrm{lik}}^{2}$, using the R package *rr2* (Ives and Li 2018; Ives 2019). The partial $R_{\mathrm{lik}}^{2}$ is calculated by comparing the full model with reduced models that remove one explanatory variable at a time and calculating the reduction in the likelihood ratio value. The $R_{\mathrm{lik}}^{2}$ corresponds to the variable excluded from the model.

We assessed multicollinearity among predictors (tropical distribution, occurrence within hotspot, species richness) computing the Variance Inflation Factors using the R package *car* (Fox and Weisberg 2019) and the Phi coefficient using the R package *psych* (Revelle 2025). VIF values lower than 2 (tropical distribution = 1.269, hotspot = 1.269, and species richness of genera = 1.000) indicated no collinearity. However, the Phi coefficient revealed a moderate correlation between tropical and hotspot variables (*rϕ* = 0.362, *p* < 0.001), consistent with their partial geographical overlap. To account for the possible non‐independence of the predictors, we considered three full model configurations set: including all four predictors simultaneously (tropical regions, hotspots, species richness of genera, and phylogeny), excluding the hotspot variable, and excluding the tropical region variable. We used the Akaike information criterion (AIC) to compare the performance of the three full model configurations, using ΔAIC < 2 and higher Akaike weights as indicators of the best‐fitting model (Table S5). Because the best fit model varied among data sets (see the Section 2.2.4 below for details on data sets and Table S5), and tropical regions and hotspots represent complementary biogeographical patterns, we retained both as biologically meaningful factors in our study.

#### 2.2.3 Spatial Autocorrelation Analysis

To explore the spatial autocorrelation component in the occurrence of SLP, we calculated the Moran's Index (Moran's *I*; Gittleman and Kot 1990). To do this, we compiled centroid data for all 368 regions using the R packages *rgeos* (Bivand et al. 2023) and *rworldmap* (South 2022), and used them to calculate the centroid for each genus distribution (see Figure 2 for centroids distribution). For genera present in various regions, we estimated the distribution centroid through the convex hull of all centroids using the function *gConvexHull* and *gCentroid* with the R package *rgeos* (Bivand et al. 2023). We used the distribution of centroids to generate a matrix of inverse distance weights and replaced the diagonal entries with zeros to avoid division by zero. We constructed a phylogenetic distance matrix from the phylogenetic tree using the R package *ape* (Paradis et al. 2004) and decomposed the phylogenetic distance matrix into a set of orthogonal eigenvectors. We computed a model with SLP as the response variable and the first 10 phylogenetic eigenvectors as explanatory variables. We took the residuals from the model and used them, along with the inverted distance matrix, to estimate Moran's *I* (see Table S7). A significant Moran's *I* value indicates that the residuals are not randomly distributed but exhibit spatial clustering.

#### 2.2.4 Rubiaceae and Non‐Rubiaceae Analyses

The family Rubiaceae includes 54% of all listed SLP genera (130 genera) and 51% of SLP genera in our analysis with the pruned phylogenetic tree (104 genera) (Table S2). To further explore the influence of this family in the observed patterns, we repeated all the above analyses (i) including only Rubiaceae genera (Rubiaceae data set hereafter) and (ii) excluding Rubiaceae genera (non‐Rubiaceae data set hereafter) (Tables S3–S7). Since we obtained similar results for the analyses of the full database applying the 50% and the 70% occurrence thresholds for classifying genera as tropical, here we only applied the original 50% distribution threshold.

Regarding the influence of our two biogeographic variables, among all the phylogenetic logistic regression models, we found that, contrary to our diversity‐driven hypothesis, for the non‐Rubiaceae data set, SLP had a negative association with occurrence within hotspots. If high species diversity promoted the random appearance of SLP, we would have expected a positive association of SLP with hotspots, where diversification is highest (Sundaram et al. 2019). To further evaluate this hypothesis, we performed a phylogenetic linear regression to test whether, as expected, species‐rich genera tend to occur inside hotspots.

## 3 Results

**FIGURE 1:**
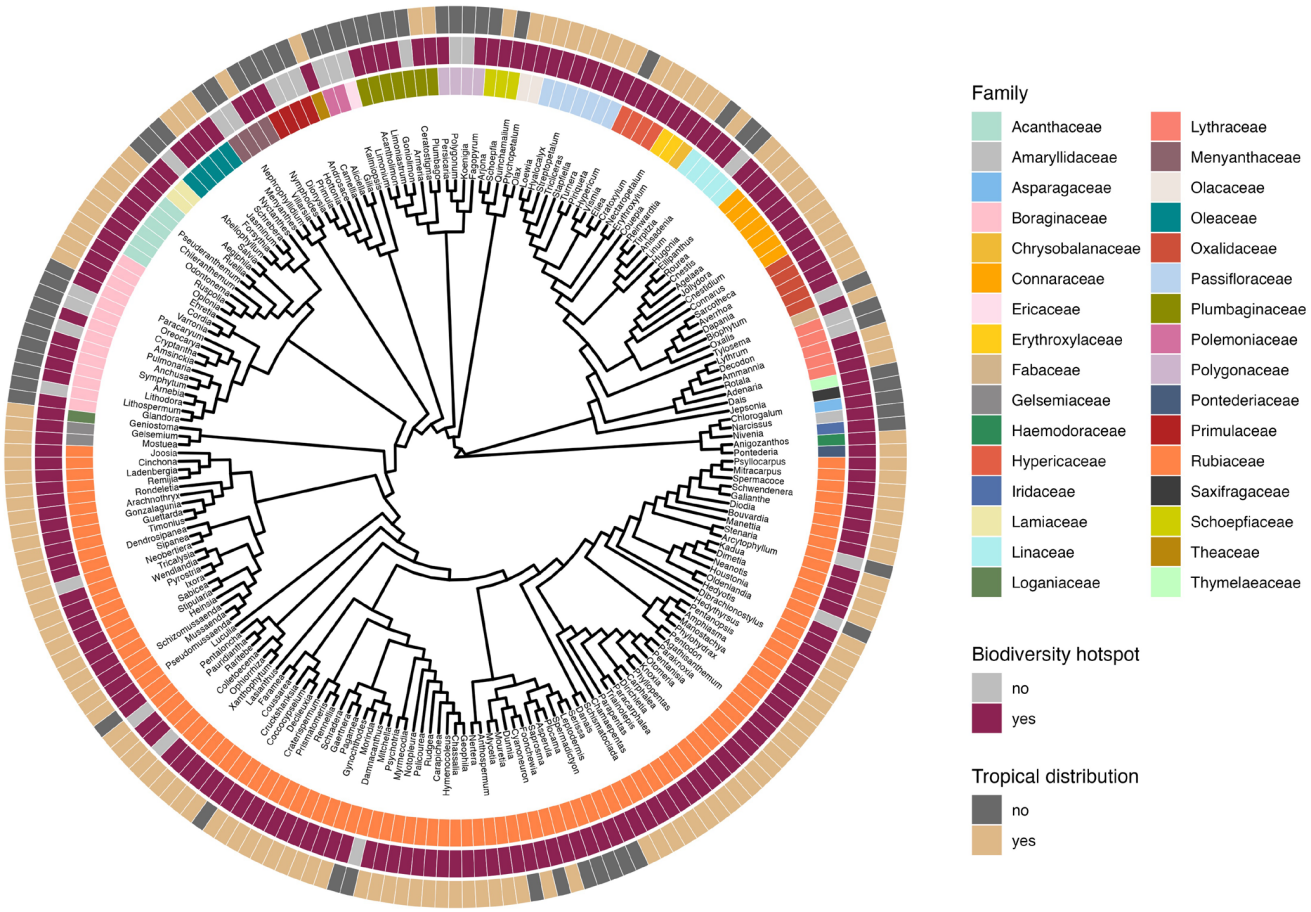
Phylogeny of style‐length polymorphic genera (SLP) with indication of their family and coding as “tropical/non‐tropical” and “within/outside hotspot.” The outermost circle represents the climate region (tropical or non‐tropical). The second circle shows their presence or absence in biodiversity hotspots. The innermost circle represents the distribution of style‐length polymorphic genera in angiosperm families. The figure includes only SLP genera. Only the topology of the phylogenetic tree is represented; it does not include branch lengths.

**FIGURE 2:**
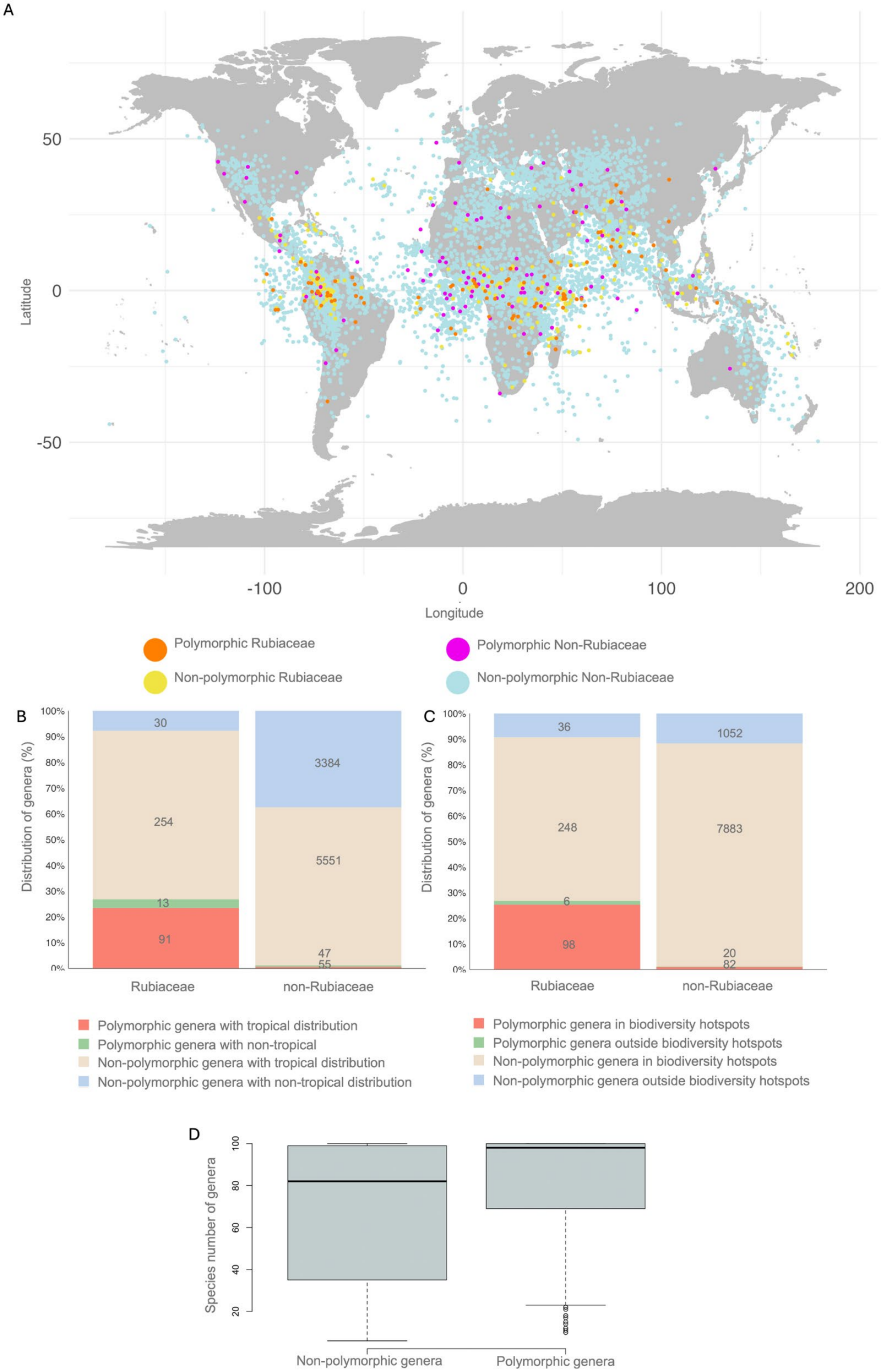
(A) Distribution of centroids of the geographic distribution of both style‐length polymorphic (SLP) and non‐polymorphic (non‐SLP) genera, separated by Rubiaceae and non‐Rubiaceae genera. Note that centroids plot in the ocean because many genera span multiple regions and/or islands. (B) Percentage of Rubiaceae and non‐Rubiaceae genera occurring within tropical regions and (C) biodiversity hotspots. The numbers inside or above the bars represent the observed number of genera within each category. (D) Number of species of SLP versus non‐SLP genera (*β* = 0.015, *z* = 5.797, *p* < 0.001). The plots were constructed using data from the genera in our final phylogeny (9425 in total, including 106 polymorphic) and with tropical classification based on the 50% threshold.

The distribution patterns of SLP are shown in Figure 1. Our exploratory analysis showed a significantly positive association of SLP with tropicality for the full data set (50% threshold: 71% of SLP genera and 63% of non‐SLP genera occur within the tropics; *χ* ^2^ = 5.077, *p* = 0.024; results for the 70% threshold: *χ* ^2^ = 10.377, *p* = 0.001), but not for either the Rubiaceae data set (*χ* ^2^ = 0.127, *p* = 0.772) nor the non‐Rubiaceae data set (*χ* ^2^ = 25.448, *p* = 0.111). There was a significant negative association between SLP and hotspots only for the non‐Rubiaceae data set (outside this family, 80% of SLP genera and 88% of non‐SLP genera occur within hotspots; *χ* ^2^ = 5.194, *p* = 0.023; results for the full data set: *χ* ^2^ = 0.000, *p* = 0.994; and for the Rubiaceae data set: *χ* ^2^ = 3.081, *p* = 0.079) (Figure 2, Table S3). SLP genera had more species than non‐SLP genera across all data sets (full data set: *t* = 6.010, *p* < 0.001; Rubiaceae data set: *t* = 4.476, *p* < 0.001; non‐Rubiaceae data set: *t* = 4.958, *p* < 0.001) (Figure 2). All the above results are from exploratory analyses using the genera included in the phylogeny (*N* = 9425 genera), which were consistent with those obtained from the initial database including the 13,266 angiosperm genera (Table S3).

The evolution of SLP showed a strong phylogenetic signal in all analyzed data sets (phylo *D* = −0.026 to 0.209, *p* resulting from no (random) phylogenetic structure = 0, *p* resulting from Brownian phylogenetic structure > 0.173) (Table S4). The occurrence of genera within the tropics and hotspots did not show phylogenetic signal, except for the analysis of the tropical distribution with the Rubiaceae data set (Table S4).

Our phylogenetic logistic regression models showed that the occurrence of SLP is mainly explained by the phylogenetic relatedness of genera (partial $R_{\mathrm{lik}}^{2}$ = 47.1%, *p* < 0.001), followed by the species richness of genera (partial $R_{\mathrm{lik}}^{2}$ = 1.8%, *p* < 0.001) (Tables S5 and S6). The occurrence of SLP did not present a significant relationship with the distribution of genera within the tropics (partial $R_{\mathrm{lik}}^{2}$ = 0.0%, *p* = 1) or biodiversity hotspots (partial $R_{\mathrm{lik}}^{2}$ = 1.2%, *p* = 0.052) (Tables S5 and S6). These results were retrieved from modeling the full data set with all four predictors and were consistent across the three model configurations (including all four predictors, excluding hotspots, and excluding tropical regions) and the three data sets analyzed (full, Rubiaceae, and non‐Rubiaceae) with two exceptions. First, in the Rubiaceae data set, the relationship between the occurrence of SLP and species richness was not significant (partial $R_{\mathrm{lik}}^{2}$ = 0.4%, *p* = 0.313). Second, in the non‐Rubiaceae data set, the occurrence of SLP showed a significant negative relationship with hotspots (partial $R_{\mathrm{lik}}^{2}$ = 1.5%, *p* < 0.001) (Table S6). For this dataset, species richness was positively related to hotspots (*β* = 11.737, *p* < 0.001).

We found no spatial autocorrelation, corrected for phylogenetic effects, in the occurrence of SLP for the full data set nor the Rubiaceae data set, but we found spatial aggregation of SLP in the non‐Rubiaceae data set (see Table S7 for statistical results).

## 4 Discussion

We investigated the phylogenetic and biogeographical determinants shaping the global distribution of one of the most outstanding outcrossing strategies in flowering plants: style‐length polymorphisms, which embody a pollinator‐dependent, specialized, and labile mating system (Simón‐Porcar et al. 2024). We tested two hypotheses to explain the global distribution of SLP in angiosperms: the tropicality‐driven hypothesis, which associates SLP with the prevalence of specialized plant‐pollinator interactions and outcrossing in tropical regions, and the diversity‐driven hypothesis, which associates the evolution of style‐length polymorphisms with high species diversity in hotspots. Although tropical regions and biodiversity hotspots showed a moderate correlation, consistent with their partial geographical overlap, all computed models (including all predictors, excluding hotspots, and excluding tropical distribution) yielded consistent results, suggesting that this correlation did not influence our main results. The effect of both geographical variables was discussed in the context of their different biological implications for SLP taxa. Our analyses provided no support for either hypothesis once phylogenetic relatedness was accounted for, suggesting instead that the occurrence of SLP is mainly influenced by the particular evolutionary history of each angiosperm lineage. The positive association between the occurrence of SLP and the species richness of genera across the full data set and the non‐Rubiaceae data set, and the negative association between the occurrence of SLP and biodiversity hotspots in non‐Rubiaceae genera, suggest that SLP may increase speciation of non‐Rubiaceae genera. Alternatively, other factors may drive both the evolution of SLP and speciation (outside hotspots in non‐Rubiaceae genera). Our spatial autocorrelation analyses revealed no spatial effects on SLP occurrence except in the non‐Rubiaceae data set, indicating that non‐Rubiaceae SLP genera are geographically more clustered than expected from their phylogenetic relatedness.

The increasing specialization of plant‐pollinator interactions towards the tropics (Olesen and Jordano 2002; Ollerton et al. 2006) has been attributed to stable climatic conditions and high diversity of biotic interactions in these regions (Pianka 1966; Hillebrand 2004; Mittelbach et al. 2007; Huntingford and Mercado 2013). This specialization, jointly with the longer life spans of tropical plants (Moeller et al. 2017), is thought to underlie the prevalence of pollinator‐mediated outcrossing in tropical biomes. We found that, overall, the occurrence of SLP was significantly associated with the tropical distribution of genera, but this pattern relied on the predominantly tropical family Rubiaceae (Davis et al. 2009), and hence the association was lost when considering phylogenic relatedness. This is not surprising, as Rubiaceae is among the most diverse families of angiosperms, and species of this family typically bear the floral phenotype that has been related to the pollination‐precision hypothesis (Simón‐Porcar et al. 2024), likely promoting the prevalence of SLP in this lineage. Interestingly, the tropical affinity of genera was phylogenetically conserved only within Rubiaceae, indicating that closely related genera in this family tend to share a predominantly tropical distribution. Although a study on the large subfamily Rubioideae showed no overall association between the presence of SLP and a tropical distribution of taxa, the loss of SLP in some clades occurred in non‐tropical areas (Ferrero et al. 2011). This might be due to the tropical origin of certain Rubiaceae clades and the time elapsed since some clades colonized non‐tropical regions.

In addition to the influence of the Rubiaceae in the tropical distribution of SLP, the prevalence of SLP in genera from other non‐tropical families could also contribute to our result. Examples include various genera in families such as Boraginaceae (the second most represented family in number of style‐polymorphic genera (~6%)), Plumbaginaceae (~3%), Linaceae (~2%), and Primulaceae (~2%), which also typically bear floral architectures that promote pollination precision. Our findings highlight the importance of taking phylogenetic relationships into account when investigating biogeographical patterns, as apparent associations may be driven by the evolutionary history of plant lineages rather than current ecological or geographic variables alone. The decoupling of SLP and tropical habitats contrasts with the predominantly tropical distribution of highly specialized pollination systems (Ollerton et al. 2006). Regarding SLP as a moderately specialized pollination system (Simón‐Porcar et al. 2024), this result suggests the value of conducting biogeographical analyses on the pollination specialization continuum (Armbruster 2014, 2017).

The lack of a positive association between the occurrence of SLP and biodiversity hotspots—regions characterized by high species richness—together with the negative relationship observed for non‐Rubiaceae genera, suggests that the occurrence of SLP is not merely a product of random processes driven by high diversification. In fact, we found that the species richness of genera is positively associated with the occurrence of SLP regardless of their occurrence inside or outside of biodiversity hotspots. This finding suggests a potential reverse causal pathway, at least for non‐Rubiaceae genera: instead of arising more frequently in already diversified lineages, SLP may actually increase species richness. Two mechanisms have been proposed in support of this hypothesis. First, as a labile reproductive trait at the genus level, the repeated gain and loss of SLP (e.g., Pérez et al. 2003; Ferrero et al. 2009, 2011) may facilitate the establishment of reproductive barriers and thus promote speciation (Rieseberg and Willis 2007). Second, SLP may enhance speciation by promoting outcrossing (Haller et al. 2014), thereby increasing the genetic diversity and adaptative potential of plant lineages (de Vos et al. 2014). Although analyses of species richness offer valuable insights (Kay and Sargent 2009) and our results embody a robust macroevolutionary pattern consistent with this hypothesis, rigorous testing at this scale will require further phylogenetic analyses to explicitly assess the effect of SLP on diversification rates. Notably, the limited number of genus‐level studies on this topic have reported contrasting results (de Vos et al. 2014; Maguilla et al. 2021), highlighting the need for state‐dependent diversification models using dated phylogenies across multiple regions and taxonomic levels (genus vs. species level). Furthermore, since most SLP plants are self‐incompatible (Ganders 1979), the potential influence of SLP on diversification could be mediated by self‐incompatibility systems (Goldberg et al. 2010; Ferrer and Good 2012), and this factor should be assessed explicitly. An alternative explanation is that other factors (e.g., dispersal mechanism, life form) may promote both the evolution of SLP and the species richness of genera, implying that the observed association is not necessarily causal.

The absence of clear biogeographic patterns in SLP occurrence at the global scale may be partly due to the variability of data within the genus level, both in terms of SLP representation and species distribution. However, this variability cannot be assessed easily at the species level, as many species also exhibit population‐level variability in SLP expression (e.g., Simón‐Porcar et al. 2015). Whilst a much more extensive compilation of species‐level data on the occurrence of SLP is far from attainable, we advocate the reconstruction of phylogenies for SLP genera across the angiosperm tree, particularly those with contrasted distribution patterns, to test whether SLP species show different associations with tropical regions or biodiversity hotspots using a meta‐analytical approach. In the meantime, we emphasize that our dataset provides comprehensive coverage of angiosperm genera, ensuring that our results are not driven by sampling biases or spurious patterns.

Our analyses found no support for the tropicality‐driven and the diversity‐driven hypotheses for the occurrence of SLP once phylogenetic relatedness was taken into account, indicating that evolutionary history is the primary driver of SLP distribution on a global scale. Our results complement the recent finding that the evolution of SLP is associated with floral traits and pollination systems related to precise pollen transfer between morphs (Simón‐Porcar et al. 2024), which remain the main known ecological driver of the macroevolutionary patterns of SLP. Thus, the evolution of this mating system appears to be shaped by the interplay between ecological factors and phylogenetic conservatism. Beyond the absence of biogeographic trends, our analyses confirm that SLP occurrence is positively associated with species richness, regardless of its underlying cause or the geographic distribution of genera.

## Author Contributions

**Letícia Rodrigues Novaes:** data curation (lead), formal analysis (lead), funding acquisition (equal), investigation (lead), methodology (equal), validation (lead), visualization (lead), writing – original draft (lead), writing – review and editing (equal). **Violeta Simón‐Porcar:** conceptualization (lead), data curation (supporting), formal analysis (supporting), funding acquisition (equal), investigation (supporting), methodology (equal), supervision (lead), validation (supporting), visualization (supporting), writing – review and editing (equal). **Rafael Molina‐Venegas:** formal analysis (supporting), methodology (equal), supervision (supporting), validation (supporting), visualization (supporting), writing – review and editing (supporting). **Juan Arroyo:** conceptualization (lead), formal analysis (supporting), funding acquisition (equal), methodology (equal), project administration (lead), supervision (lead), validation (supporting), visualization (supporting), writing – review and editing (equal).

## Funding

This publication is part of the project PID2024‐161829NB‐I00, funded by MICIU/AEI/ 10.13039/501100011033 and by ERDF/EU.This project has received funding from the European Union’s Horizon 2020 research and innovation programme under grant agreement no. 897890. The present work was funded by the projects PID2024‐161829NB‐I00, PGC2018‐099608‐B‐I00 and PID2021‐122715NB‐I00 funded by MICIU /AEI /10.13039/501100011033 / FEDER, UE, granted to J.A and V.S.P. Additional funding was provided by the MICINN through an FPU fellowship (FPU20/05843; L.R.N.).

## Conflicts of Interest

The authors declare no conflicts of interest.

## Supporting information


**Appendix S1:** ece372659‐sup‐0001‐AppendixS1.docx.

## Data Availability

The datasets supporting this study are available on the Dryad digital repository. Dataset: https://doi.org/10.5061/dryad.t76hdr8cq.
