# Supplementary material for: A Global Analysis of the Distribution Patterns of Style‐Length Polymorphisms Across Angiosperms
Source: Ecol Evol. 2026 Apr 20;16(4):e72659. doi: 10.1002/ece3.72659 (PMC13096687; doi:10.1002/ece3.72659)
Supplement: Supplementary file 1 — Appendix S1: ece372659‐sup‐0001‐AppendixS1.docx. [file ECE3-16-e72659-s001.docx]

## **Supporting information**

**Table S1.** List of all 368 regions from Plants of the World Online (POWO) with those occurring within tropical regions or biodiversity hotspots coded as 1, and all others coded as 0.

| All Regions | Tropical regions | Biodiversity hotspots |
| --- | --- | --- |
| Alberta_ABT | 0 | 0 |
| Afghanistan_AFG | 0 | 1 |
| ArgentinaNortheast_AGE | 0 | 0 |
| ArgentinaSouth_AGS | 0 | 0 |
| ArgentinaNorthwest_AGW | 0 | 1 |
| Alabama_ALA | 0 | 0 |
| Albania_ALB | 0 | 1 |
| Aldabra_ALD | 1 | 1 |
| Algeria_ALG | 1 | 1 |
| Altay_ALT | 0 | 0 |
| AleutianIs_ALU | 0 | 0 |
| Amur_AMU | 0 | 0 |
| AndamanIs_AND | 1 | 1 |
| Angola_ANG | 1 | 0 |
| Antarctica_ANT | 0 | 0 |
| Arizona_ARI | 0 | 1 |
| Arkansas_ARK | 0 | 0 |
| Aruba_ARU | 1 | 1 |
| Ascension_ASC | 1 | 1 |
| Alaska_ASK | 0 | 0 |
| AmsterdamStPaulIs_ASP | 0 | 0 |
| Assam_ASS | 0 | 1 |
| AntipodeanIs_ATP | 0 | 1 |
| Austria_AUT | 0 | 0 |
| Azores_AZO | 0 | 1 |
| Bahamas_BAH | 1 | 1 |
| Baleares_BAL | 0 | 1 |
| Bangladesh_BAN | 1 | 0 |
| Benin_BEN | 1 | 0 |
| Bermuda_BER | 0 | 1 |
| Belgium_BGM | 0 | 0 |
| BismarckArchipelago_BIS | 1 | 1 |
| Burkina_BKN | 1 | 0 |
| Belarus_BLR | 0 | 0 |
| BalticStates_BLT | 0 | 0 |
| Belize_BLZ | 1 | 1 |
| Bolivia_BOL | 1 | 1 |
| Borneo_BOR | 1 | 1 |
| Botswana_BOT | 1 | 0 |
| BritishColumbia_BRC | 0 | 0 |
| Buryatiya_BRY | 0 | 0 |
| Bulgaria_BUL | 0 | 0 |
| Burundi_BUR | 1 | 1 |
| BrazilWestCentral_BZC | 1 | 1 |
| BrazilNortheast_BZE | 1 | 1 |
| BrazilSoutheast_BZL | 1 | 1 |
| BrazilNorth_BZN | 1 | 0 |
| BrazilSouth_BZS | 1 | 1 |
| Cabinda_CAB | 1 | 0 |
| CentralAfricanRepublic_CAF | 1 | 0 |
| California_CAL | 0 | 1 |
| CaymanIs_CAY | 1 | 1 |
| Cambodia_CBD | 1 | 1 |
| ChagosArchipelago_CGS | 1 | 1 |
| Chad_CHA | 1 | 0 |
| ChinaSouthCentral_CHC | 0 | 1 |
| Hainan_CHH | 1 | 1 |
| InnerMongolia_CHI | 0 | 0 |
| Manchuria_CHM | 0 | 0 |
| ChinaNorthCentral_CHN | 0 | 0 |
| Qinghai_CHQ | 0 | 1 |
| ChinaSoutheast_CHS | 0 | 1 |
| Tibet_CHT | 0 | 1 |
| Xinjiang_CHX | 0 | 0 |
| CocosKeelingIs_CKI | 1 | 1 |
| ChileCentral_CLC | 0 | 1 |
| Colombia_CLM | 1 | 1 |
| ChileNorth_CLN | 1 | 1 |
| ChileSouth_CLS | 0 | 1 |
| Cameroon_CMN | 1 | 1 |
| Connecticut_CNT | 0 | 0 |
| CanariasIs_CNY | 0 | 1 |
| Colorado_COL | 0 | 0 |
| Comoros_COM | 1 | 1 |
| Congo_CON | 1 | 0 |
| CookIs_COO | 1 | 1 |
| Corse_COR | 0 | 1 |
| CostaRica_COS | 1 | 1 |
| CAmericanPacificIs_CPI | 1 | 1 |
| CapeProvinces_CPP | 0 | 1 |
| CapriviStrip_CPV | 1 | 0 |
| CarolineIs_CRL | 1 | 1 |
| CrozetIs_CRZ | 0 | 1 |
| Chita_CTA | 0 | 0 |
| ChatamIs_CTM | 0 | 1 |
| Cuba_CUB | 1 | 1 |
| CapeVerde_CVI | 1 | 1 |
| Cyprus_CYP | 0 | 1 |
| Czechoslovakia_CZE | 0 | 0 |
| Delaware_DEL | 0 | 0 |
| Denmark_DEN | 0 | 0 |
| Djibouti_DJI | 1 | 1 |
| DominicanRepublic_DOM | 1 | 1 |
| DesventuradosIs_DSV | 0 | 1 |
| EastAegeanIs_EAI | 0 | 1 |
| EasterIs_EAS | 0 | 1 |
| Ecuador_ECU | 1 | 1 |
| Egypt_EGY | 1 | 0 |
| EastHimalaya_EHM | 0 | 1 |
| ElSalvador_ELS | 1 | 1 |
| EquatorialGuinea_EQG | 1 | 1 |
| Eritrea_ERI | 1 | 1 |
| Ethiopia_ETH | 1 | 1 |
| FalklandIs_FAL | 0 | 1 |
| Fiji_FIJ | 1 | 1 |
| Finland_FIN | 0 | 0 |
| Florida_FLA | 0 | 0 |
| Foroyar_FOR | 0 | 0 |
| France_FRA | 0 | 1 |
| FrenchGuiana_FRG | 1 | 0 |
| Gabon_GAB | 1 | 0 |
| Galapagos_GAL | 1 | 1 |
| GambiaThe_GAM | 1 | 0 |
| Georgia_GEO | 0 | 1 |
| Germany_GER | 0 | 0 |
| GulfofGuineaIs_GGI | 1 | 1 |
| Ghana_GHA | 1 | 1 |
| GilbertIs_GIL | 1 | 1 |
| GuineaBissau_GNB | 1 | 0 |
| Greenland_GNL | 0 | 0 |
| GreatBritain_GRB | 0 | 0 |
| Greece_GRC | 0 | 1 |
| GulfStates_GST | 1 | 1 |
| Guatemala_GUA | 1 | 1 |
| Guinea_GUI | 1 | 1 |
| Guyana_GUY | 1 | 0 |
| Haiti_HAI | 1 | 1 |
| Hawaii_HAW | 1 | 1 |
| HowlandBakerIs_HBI | 1 | 1 |
| HeardMcDonaldIs_HMD | 0 | 0 |
| Honduras_HON | 1 | 1 |
| Hungary_HUN | 0 | 0 |
| Iceland_ICE | 0 | 0 |
| Idaho_IDA | 0 | 0 |
| Illinois_ILL | 0 | 0 |
| India_IND | 1 | 1 |
| Indiana_INI | 0 | 0 |
| Iowa_IOW | 0 | 0 |
| Ireland_IRE | 0 | 0 |
| Irkutsk_IRK | 0 | 0 |
| Iran_IRN | 0 | 1 |
| Iraq_IRQ | 0 | 1 |
| Italia_ITA | 0 | 1 |
| IvoryCoast_IVO | 1 | 1 |
| Jamaica_JAM | 1 | 1 |
| Japan_JAP | 0 | 1 |
| Jawa_JAW | 1 | 1 |
| JuanFernandezIs_JNF | 0 | 1 |
| Kamchatka_KAM | 0 | 0 |
| Kansas_KAN | 0 | 0 |
| Kazakhstan_KAZ | 0 | 1 |
| Kerguelen_KEG | 0 | 1 |
| Kenya_KEN | 1 | 1 |
| KermadecIs_KER | 0 | 1 |
| Kirgizstan_KGZ | 0 | 1 |
| Khabarovsk_KHA | 0 | 0 |
| Korea_KOR | 0 | 0 |
| Krasnoyarsk_KRA | 0 | 1 |
| Kriti_KRI | 0 | 1 |
| Krym_KRY | 0 | 1 |
| Kentucky_KTY | 0 | 0 |
| KurilIs_KUR | 0 | 1 |
| Kuwait_KUW | 0 | 0 |
| Kazanretto_KZN | 0 | 1 |
| Labrador_LAB | 0 | 0 |
| Laos_LAO | 1 | 1 |
| Liberia_LBR | 1 | 1 |
| LebanonSyria_LBS | 0 | 1 |
| Libya_LBY | 1 | 0 |
| LaccadiveIs_LDV | 1 | 1 |
| LeewardIs_LEE | 1 | 1 |
| Lesotho_LES | 0 | 0 |
| LineIs_LIN | 1 | 1 |
| Louisiana_LOU | 0 | 0 |
| LesserSundaIs_LSI | 1 | 1 |
| Magadan_MAG | 0 | 0 |
| Maine_MAI | 0 | 0 |
| Manitoba_MAN | 0 | 0 |
| MacquarieIs_MAQ | 0 | 1 |
| Massachusetts_MAS | 0 | 0 |
| Mauritius_MAU | 1 | 1 |
| MozambiqueChannelIs_MCI | 1 | 1 |
| MarcusI_MCS | 1 | 1 |
| Madagascar_MDG | 1 | 1 |
| Madeira_MDR | 0 | 1 |
| Maldives_MDV | 1 | 0 |
| Michigan_MIC | 0 | 0 |
| Minnesota_MIN | 0 | 0 |
| Mali_MLI | 1 | 0 |
| Malawi_MLW | 1 | 1 |
| Malaya_MLY | 1 | 1 |
| Montana_MNT | 0 | 0 |
| Maluku_MOL | 1 | 1 |
| Mongolia_MON | 0 | 0 |
| Morocco_MOR | 0 | 1 |
| Mozambique_MOZ | 1 | 1 |
| MarionPrinceEdwardIs_MPE | 0 | 0 |
| Marianas_MRN | 1 | 1 |
| Marquesas_MRQ | 1 | 1 |
| MarshallIs_MRS | 1 | 0 |
| Maryland_MRY | 0 | 1 |
| Mississippi_MSI | 0 | 0 |
| Missouri_MSO | 0 | 0 |
| Mauritania_MTN | 1 | 0 |
| MexicoCentral_MXC | 1 | 1 |
| MexicoNortheast_MXE | 0 | 1 |
| MexicoGulf_MXG | 1 | 1 |
| MexicanPacificIs_MXI | 1 | 1 |
| MexicoNorthwest_MXN | 0 | 1 |
| MexicoSouthwest_MXS | 1 | 1 |
| MexicoSoutheast_MXT | 1 | 1 |
| Myanmar_MYA | 1 | 1 |
| Namibia_NAM | 1 | 1 |
| KwaZuluNatal_NAT | 0 | 1 |
| NewBrunswick_NBR | 0 | 0 |
| NorthCarolina_NCA | 0 | 0 |
| NicobarIs_NCB | 1 | 1 |
| NorthCaucasus_NCS | 0 | 1 |
| NorthDakota_NDA | 0 | 0 |
| Nebraska_NEB | 0 | 0 |
| Nepal_NEP | 0 | 1 |
| Netherlands_NET | 0 | 0 |
| Nevada_NEV | 0 | 0 |
| NorfolkIs_NFK | 0 | 1 |
| Newfoundland_NFL | 0 | 0 |
| Nigeria_NGA | 1 | 1 |
| Niger_NGR | 1 | 0 |
| Nicaragua_NIC | 1 | 1 |
| NetherlandsAntilles_NLA | 1 | 1 |
| NanseiShoto_NNS | 0 | 1 |
| Norway_NOR | 0 | 0 |
| Nauru_NRU | 1 | 1 |
| NovaScotia_NSC | 0 | 0 |
| NewSouthWales_NSW | 0 | 1 |
| NorthernTerritory_NTA | 1 | 0 |
| Niue_NUE | 1 | 1 |
| Nunavut_NUN | 0 | 0 |
| NewCaledonia_NWC | 1 | 1 |
| NewGuinea_NWG | 1 | 0 |
| NewHampshire_NWH | 0 | 0 |
| NewJersey_NWJ | 0 | 0 |
| NewMexico_NWM | 0 | 0 |
| NorthwestTerritories_NWT | 0 | 0 |
| NewYork_NWY | 0 | 0 |
| NewZealandNorth_NZN | 0 | 1 |
| NewZealandSouth_NZS | 0 | 1 |
| FreeState_OFS | 0 | 1 |
| OgasawaraShoto_OGA | 0 | 1 |
| Ohio_OHI | 0 | 0 |
| Oklahoma_OKL | 0 | 0 |
| Oman_OMA | 1 | 1 |
| Ontario_ONT | 0 | 0 |
| Oregon_ORE | 0 | 0 |
| Pakistan_PAK | 0 | 1 |
| Palestine_PAL | 0 | 0 |
| Panama_PAN | 1 | 1 |
| Paraguay_PAR | 1 | 1 |
| PrinceEdwardI_PEI | 0 | 0 |
| Pennsylvania_PEN | 0 | 0 |
| Peru_PER | 1 | 1 |
| Philippines_PHI | 1 | 1 |
| PhoenixIs_PHX | 1 | 1 |
| PitcairnIs_PIT | 0 | 1 |
| Poland_POL | 0 | 0 |
| Portugal_POR | 0 | 1 |
| Primorye_PRM | 0 | 0 |
| PuertoRico_PUE | 1 | 1 |
| Queensland_QLD | 1 | 1 |
| Quebec_QUE | 0 | 0 |
| Reunion_REU | 1 | 1 |
| RhodeIsland_RHO | 0 | 0 |
| Rodrigues_ROD | 1 | 0 |
| Romania_ROM | 0 | 0 |
| CentralEuropeanRussia_RUC | 0 | 0 |
| EastEuropeanRussia_RUE | 0 | 0 |
| NorthEuropeanRussia_RUN | 0 | 0 |
| SouthEuropeanRussia_RUS | 0 | 1 |
| NorthwestEuropeanRussia_RUW | 0 | 0 |
| Rwanda_RWA | 1 | 1 |
| Sakhalin_SAK | 0 | 0 |
| Samoa_SAM | 1 | 1 |
| Sardegna_SAR | 0 | 1 |
| Saskatchewan_SAS | 0 | 0 |
| SaudiArabia_SAU | 1 | 1 |
| SouthCarolina_SCA | 0 | 0 |
| SocietyIs_SCI | 1 | 1 |
| SouthChinaSea_SCS | 1 | 1 |
| SantaCruzIs_SCZ | 1 | 1 |
| SouthDakota_SDA | 0 | 0 |
| Selvagens_SEL | 0 | 1 |
| Senegal_SEN | 1 | 0 |
| Seychelles_SEY | 1 | 1 |
| SouthGeorgia_SGE | 0 | 0 |
| Sicilia_SIC | 0 | 1 |
| SierraLeone_SIE | 1 | 1 |
| Sinai_SIN | 0 | 1 |
| SouthAustralia_SOA | 0 | 0 |
| Socotra_SOC | 1 | 1 |
| SolomonIs_SOL | 1 | 1 |
| Somalia_SOM | 1 | 1 |
| Spain_SPA | 0 | 1 |
| SriLanka_SRL | 1 | 1 |
| SouthSandwichIs_SSA | 0 | 1 |
| StHelena_STH | 1 | 1 |
| Sudan_SUD | 1 | 1 |
| Sulawesi_SUL | 1 | 1 |
| Sumatera_SUM | 1 | 1 |
| Suriname_SUR | 1 | 0 |
| Svalbard_SVA | 0 | 0 |
| SouthwestCaribbean_SWC | 1 | 1 |
| Sweden_SWE | 0 | 0 |
| Switzerland_SWI | 0 | 0 |
| Swaziland_SWZ | 0 | 1 |
| Taiwan_TAI | 1 | 0 |
| Tanzania_TAN | 1 | 1 |
| Tasmania_TAS | 0 | 0 |
| TurksCaicosIs_TCI | 1 | 1 |
| Transcaucasus_TCS | 0 | 1 |
| TristanDaCunha_TDC | 0 | 1 |
| Tennessee_TEN | 0 | 0 |
| Texas_TEX | 0 | 0 |
| Thailand_THA | 1 | 1 |
| Turkmenistan_TKM | 0 | 1 |
| Togo_TOG | 1 | 1 |
| TokelauManihiki_TOK | 1 | 1 |
| Tonga_TON | 1 | 1 |
| TrinidadTobago_TRT | 1 | 1 |
| Tuamotu_TUA | 1 | 1 |
| TubuaiIs_TUB | 1 | 1 |
| TurkeyInEuropo_TUE | 0 | 1 |
| Tunisia_TUN | 0 | 1 |
| Turkey_TUR | 0 | 1 |
| Tuvalu_TUV | 1 | 1 |
| Tuva_TVA | 0 | 0 |
| NorthernProvinces_TVL | 0 | 1 |
| Tadzhikistan_TZK | 0 | 1 |
| Uganda_UGA | 1 | 1 |
| Ukraine_UKR | 0 | 0 |
| Uruguay_URU | 0 | 0 |
| Utah_UTA | 0 | 0 |
| Uzbekistan_UZB | 0 | 1 |
| Vanuatu_VAN | 1 | 1 |
| Venezuela_VEN | 1 | 1 |
| Vermont_VER | 0 | 0 |
| Victoria_VIC | 0 | 1 |
| Vietnam_VIE | 1 | 1 |
| VenezuelanAntilles_VNA | 1 | 1 |
| Virginia_VRG | 0 | 0 |
| WakeI_WAK | 1 | 1 |
| WallisFutunaIs_WAL | 1 | 1 |
| Washington_WAS | 0 | 0 |
| WesternAustralia_WAU | 1 | 1 |
| DistrictofColumbia_WDC | 0 | 0 |
| WestHimalaya_WHM | 0 | 1 |
| WindwardIs_WIN | 0 | 1 |
| Wisconsin_WIS | 0 | 0 |
| WesternSahara_WSA | 1 | 0 |
| WestSiberia_WSB | 0 | 0 |
| WestVirginia_WVA | 0 | 0 |
| Wyoming_WYO | 0 | 0 |
| ChristmasI_XMS | 1 | 1 |
| Yakutiya_YAK | 0 | 0 |
| Yemen_YEM | 1 | 1 |
| Yugoslavia_YUG | 0 | 1 |
| Yukon_YUK | 0 | 0 |
| Zaire_ZAI | 1 | 0 |
| Zambia_ZAM | 1 | 1 |
| Zimbabwe_ZIM | 1 | 0 |

**Table S2.** List of style-length polymorphic genera from Simón-Porcar et al. 2024, with an indication of their family and their inclusion in our analyses. The analyses in this study included 206 of the 243 SLP genera (85% of the SLP genera from the original database of Simón-Porcar et al. 2024). Genera were excluded due to a lack of distribution data.

| Genus | Family | Included in the analyses |
| --- | --- | --- |
| *Chileranthemum* | Acanthaceae | yes |
| *Odontonema* | Acanthaceae | yes |
| *Oplonia* | Acanthaceae | yes |
| *Pseuderanthemum* | Acanthaceae | yes |
| *Ruellia* | Acanthaceae | yes |
| *Ruspolia* | Acanthaceae | yes |
| *Narcissus* | Amaryllidaceae | yes |
| *Chlorogalum* | Asparagaceae | yes |
| *Amsinckia* | Boraginaceae | yes |
| *Anchusa* | Boraginaceae | yes |
| *Arnebia* | Boraginaceae | yes |
| *Cordia* | Boraginaceae | yes |
| *Cryptantha* | Boraginaceae | yes |
| *Ehretia* | Boraginaceae | yes |
| *Glandora* | Boraginaceae | yes |
| *Lithodora* | Boraginaceae | yes |
| *Lithospermum* | Boraginaceae | yes |
| *Oreocarya* | Boraginaceae | yes |
| *Paracaryum* | Boraginaceae | yes |
| *Pulmonaria* | Boraginaceae | yes |
| *Symphytum* | Boraginaceae | yes |
| *Varronia* | Boraginaceae | yes |
| *Silvianthus* | Carlemanniaceae | no |
| *Couepia* | Chrysobalanaceae | yes |
| *Agelaea* | Connaraceae | yes |
| *Burttia* | Connaraceae | no |
| *Cnestidium* | Connaraceae | yes |
| *Cnestis* | Connaraceae | yes |
| *Connarus* | Connaraceae | yes |
| *Ellipanthus* | Connaraceae | yes |
| *Jollydora* | Connaraceae | yes |
| *Pseudoconnarus* | Connaraceae | no |
| *Rourea* | Connaraceae | yes |
| *Vismianthus* | Connaraceae | no |
| *Epacris* | Ericaceae | no |
| *Kalmiopsis* | Ericaceae | yes |
| *Erythroxylum* | Erythroxylaceae | yes |
| *Nectaropetalum* | Erythroxylaceae | yes |
| *Tylosema* | Fabaceae | yes |
| *Gelsemium* | Gelsemiaceae | yes |
| *Mostuea* | Gelsemiaceae | yes |
| *Curtia* | Gentianaceae | no |
| *Exochaenium* | Gentianaceae | no |
| *Hockinia* | Gentianaceae | no |
| *Sebaea* | Gentianaceae | no |
| *Tapeinostemon* | Gentianaceae | no |
| *Voyriella* | Gentianaceae | no |
| *Anigozanthos* | Haemodoraceae | yes |
| *Cratoxylum* | Hypericaceae | yes |
| *Eliea* | Hypericaceae | yes |
| *Hypericum* | Hypericaceae | yes |
| *Vismia* | Hypericaceae | yes |
| *Nivenia* | Iridaceae | yes |
| *Aegiphila* | Lamiaceae | yes |
| *Salvia* | Lamiaceae | yes |
| *Anisadenia* | Linaceae | yes |
| *Hugonia* | Linaceae | yes |
| *Linum* | Linaceae | yes |
| *Reinwardtia* | Linaceae | yes |
| *Tirpitzia* | Linaceae | yes |
| *Geniostoma* | Loganiaceae | yes |
| *Adenaria* | Lythraceae | yes |
| *Ammannia* | Lythraceae | yes |
| *Decodon* | Lythraceae | yes |
| *Lythrum* | Lythraceae | yes |
| *Pemphis* | Lythraceae | no |
| *Rotala* | Lythraceae | yes |
| *Melochia* | Malvaceae | no |
| *Waltheria* | Malvaceae | no |
| *Menyanthes* | Menyanthaceae | yes |
| *Nephrophyllidium* | Menyanthaceae | yes |
| *Nymphoides* | Menyanthaceae | yes |
| *Villarsia* | Menyanthaceae | yes |
| *Olax* | Olacaceae | yes |
| *Ptychopetalum* | Olacaceae | yes |
| *Abeliophyllum* | Oleaceae | yes |
| *Forsythia* | Oleaceae | yes |
| *Jasminum* | Oleaceae | yes |
| *Nyctanthes* | Oleaceae | yes |
| *Schrebera* | Oleaceae | yes |
| *Averrhoa* | Oxalidaceae | yes |
| *Biophytum* | Oxalidaceae | yes |
| *Dapania* | Oxalidaceae | yes |
| *Oxalis* | Oxalidaceae | yes |
| *Sarcotheca* | Oxalidaceae | yes |
| *Hyalocalyx* | Passifloraceae | yes |
| *Loewia* | Passifloraceae | yes |
| *Piriqueta* | Passifloraceae | yes |
| *Stapfiella* | Passifloraceae | yes |
| *Streptopetalum* | Passifloraceae | yes |
| *Tricliceras* | Passifloraceae | yes |
| *Turnera* | Passifloraceae | yes |
| *Acantholimon* | Plumbaginaceae | yes |
| *Armeria* | Plumbaginaceae | yes |
| *Ceratostigma* | Plumbaginaceae | yes |
| *Goniolimon* | Plumbaginaceae | yes |
| *Limoniastrum* | Plumbaginaceae | yes |
| *Limonium* | Plumbaginaceae | yes |
| *Plumbago* | Plumbaginaceae | yes |
| *Aliciella* | Polemoniaceae | yes |
| *Gilia* | Polemoniaceae | yes |
| *Fagopyrum* | Polygonaceae | yes |
| *Koenigia* | Polygonaceae | yes |
| *Oxygonum* | Polygonaceae | no |
| *Persicaria* | Polygonaceae | yes |
| *Polygonum* | Polygonaceae | yes |
| *Pontederia* | Pontederiaceae | yes |
| *Androsace* | Primulaceae | yes |
| *Dionysia* | Primulaceae | yes |
| *Hottonia* | Primulaceae | yes |
| *Primula* | Primulaceae | yes |
| *Agathisanthemum* | Rubiaceae | yes |
| *Airosperma* | Rubiaceae | no |
| *Amphiasma* | Rubiaceae | yes |
| *Anthorrhiza* | Rubiaceae | no |
| *Anthospermum* | Rubiaceae | yes |
| *Arachnothryx* | Rubiaceae | yes |
| *Arcytophyllum* | Rubiaceae | yes |
| *Asperula* | Rubiaceae | yes |
| *Batopedina* | Rubiaceae | no |
| *Bouvardia* | Rubiaceae | yes |
| *Bradea* | Rubiaceae | no |
| *Carapichea* | Rubiaceae | yes |
| *Carphalea* | Rubiaceae | yes |
| *Chamaepentas* | Rubiaceae | yes |
| *Chassalia* | Rubiaceae | yes |
| *Cinchona* | Rubiaceae | yes |
| *Coccocypselum* | Rubiaceae | yes |
| *Colletoecema* | Rubiaceae | yes |
| *Coussarea* | Rubiaceae | yes |
| *Craterispermum* | Rubiaceae | yes |
| *Cruckshanksia* | Rubiaceae | yes |
| *Cyanoneuron* | Rubiaceae | yes |
| *Damnacanthus* | Rubiaceae | yes |
| *Danais* | Rubiaceae | yes |
| *Declieuxia* | Rubiaceae | yes |
| *Dendrosipanea* | Rubiaceae | yes |
| *Dibrachionostylus* | Rubiaceae | yes |
| *Dimetia* | Rubiaceae | yes |
| *Diodia* | Rubiaceae | yes |
| *Dirichletia* | Rubiaceae | yes |
| *Dunnia* | Rubiaceae | yes |
| *Faramea* | Rubiaceae | yes |
| *Foonchewia* | Rubiaceae | yes |
| *Gaertnera* | Rubiaceae | yes |
| *Galianthe* | Rubiaceae | yes |
| *Geophila* | Rubiaceae | yes |
| *Gonzalagunia* | Rubiaceae | yes |
| *Guettarda* | Rubiaceae | yes |
| *Gynochthodes* | Rubiaceae | yes |
| *Hedyotis* | Rubiaceae | yes |
| *Hedythyrsus* | Rubiaceae | yes |
| *Heinsia* | Rubiaceae | yes |
| *Hindsia* | Rubiaceae | no |
| *Houstonia* | Rubiaceae | yes |
| *Hydnophytum* | Rubiaceae | no |
| *Hymenocoleus* | Rubiaceae | yes |
| *Involucrella* | Rubiaceae | no |
| *Ixora* | Rubiaceae | yes |
| *Joosia* | Rubiaceae | yes |
| *Kadua* | Rubiaceae | yes |
| *Knoxia* | Rubiaceae | yes |
| *Ladenbergia* | Rubiaceae | yes |
| *Lasianthus* | Rubiaceae | yes |
| *Lathraeocarpa* | Rubiaceae | no |
| *Lelya* | Rubiaceae | no |
| *Leptodermis* | Rubiaceae | yes |
| *Leptomischus* | Rubiaceae | no |
| *Luculia* | Rubiaceae | yes |
| *Machaonia* | Rubiaceae | no |
| *Manettia* | Rubiaceae | yes |
| *Manostachya* | Rubiaceae | yes |
| *Mitchella* | Rubiaceae | yes |
| *Mitracarpus* | Rubiaceae | yes |
| *Morinda* | Rubiaceae | yes |
| *Mouretia* | Rubiaceae | yes |
| *Mussaenda* | Rubiaceae | yes |
| *Mycetia* | Rubiaceae | yes |
| *Myrmecodia* | Rubiaceae | yes |
| *Myrmephytum* | Rubiaceae | no |
| *Neanotis* | Rubiaceae | yes |
| *Neobertiera* | Rubiaceae | yes |
| *Neomussaenda* | Rubiaceae | no |
| *Nertera* | Rubiaceae | yes |
| *Notopleura* | Rubiaceae | yes |
| *Oldenlandia* | Rubiaceae | yes |
| *Ophiorrhiza* | Rubiaceae | yes |
| *Otomeria* | Rubiaceae | yes |
| *Pagamea* | Rubiaceae | yes |
| *Palicourea* | Rubiaceae | yes |
| *Paracarphalea* | Rubiaceae | yes |
| *Paraknoxia* | Rubiaceae | yes |
| *Parapentas* | Rubiaceae | yes |
| *Pauridiantha* | Rubiaceae | yes |
| *Pentaloncha* | Rubiaceae | yes |
| *Pentanisia* | Rubiaceae | yes |
| *Pentanopsis* | Rubiaceae | yes |
| *Pentas* | Rubiaceae | no |
| *Pentodon* | Rubiaceae | yes |
| *Perama* | Rubiaceae | no |
| *Phyllopentas* | Rubiaceae | yes |
| *Phylohydrax* | Rubiaceae | yes |
| *Plocama* | Rubiaceae | yes |
| *Prismatomeris* | Rubiaceae | yes |
| *Pseudomussaenda* | Rubiaceae | yes |
| *Pseudopyxis* | Rubiaceae | no |
| *Psychotria* | Rubiaceae | yes |
| *Psyllocarpus* | Rubiaceae | yes |
| *Pteridocalyx* | Rubiaceae | no |
| *Pyrostria* | Rubiaceae | yes |
| *Raritebe* | Rubiaceae | yes |
| *Remijia* | Rubiaceae | yes |
| *Rennellia* | Rubiaceae | yes |
| *Rondeletia* | Rubiaceae | yes |
| *Rudgea* | Rubiaceae | yes |
| *Sabicea* | Rubiaceae | yes |
| *Sacosperma* | Rubiaceae | no |
| *Saprosma* | Rubiaceae | yes |
| *Schismatoclada* | Rubiaceae | yes |
| *Schizomussaenda* | Rubiaceae | yes |
| *Schradera* | Rubiaceae | yes |
| *Schwendenera* | Rubiaceae | yes |
| *Serissa* | Rubiaceae | yes |
| *Sipanea* | Rubiaceae | yes |
| *Spermacoce* | Rubiaceae | yes |
| *Spermadictyon* | Rubiaceae | yes |
| *Stenaria* | Rubiaceae | yes |
| *Stenotis* | Rubiaceae | no |
| *Stephanococcus* | Rubiaceae | no |
| *Stipularia* | Rubiaceae | yes |
| *Temnopteryx* | Rubiaceae | no |
| *Timonius* | Rubiaceae | yes |
| *Tobagoa* | Rubiaceae | no |
| *Tortuella* | Rubiaceae | no |
| *Triainolepis* | Rubiaceae | yes |
| *Tricalysia* | Rubiaceae | yes |
| *Wendlandia* | Rubiaceae | yes |
| *Xanthophytum* | Rubiaceae | yes |
| *Jepsonia* | Saxifragaceae | yes |
| *Arjona* | Schoepfiaceae | yes |
| *Quinchamalium* | Schoepfiaceae | yes |
| *Schoepfia* | Schoepfiaceae | yes |
| *Camellia* | Theaceae | yes |
| *Dais* | Thymelaeaceae | yes |

**Table S3.** Statistical results of the exploratory analyses (chi-square test and t-test). Polymorphic genera with tropical distribution (Poly-TR); Polymorphic genera with non-tropical distribution (Poly-Non-TR)**;** Non-polymorphic genera with tropical distribution (Non-poly-TR); Non-polymorphic genera with non-tropical distribution (Non-poly-Non-TR)**;** Polymorphic genera in biodiversity hotspots (Poly-HS); Polymorphic genera outside biodiversity hotspots (Poly-Non-HS); Non-polymorphic genera in biodiversity hotspots (Non-poly-HS); Non-polymorphic genera outside biodiversity hotspots (Non-poly-Non-HS).

| **(A)ALL GENERA OF OUR INITIAL PHYLOGENY**  **(13,266 GENERA)** | | | | | | |  |  |  |  |  |  |  |  |  |  |  |  |
| --- | --- | --- | --- | --- | --- | --- | --- | --- | --- | --- | --- | --- | --- | --- | --- | --- | --- | --- |
|  | Tropical distribution | | | | | | | | Biodiversity hotspots | | | | | | Number of species of the genera | | | |
|  | Poly-TR | Poly-Non-TR | Non-poly-TR | Non-poly-Non-TR | X^2^ | p-value | | | Poly-HS | Poly-Non-HS | Non-poly-HS | Non-poly-Non-HS | X^2^ | p-value | Number of species polymorphic genera (mean ± SD) | Number of species non-polymorphic genera (mean ± SD) | t-student | p-value |
| Full data set using a 50% threshold for tropical distribution | 180 (1.4%) | 63 (0.5%) | 8585 (65%) | 4438 (34%) | 67.132 | **0.009*** | | | 214 (1.6%) | 29 (0.2%) | 11469 (87%) | 1554 (12%) | 0.000 | 1.000 | 77.25 ± 30.67 | 61.08 ± 35.10 | 7.130 | **<0.001*** |
| Full data set using a 70% threshold for tropical distribution | 164 (1.3%) | 79 (0.6%) | 7306 (55%) | 5717 (43%) | 12.118 | **<0.001*** | | | / | / | / | / | / | / | / | / | / | / |
| Rubiaceae data set | 115 (20%) | 15 (2.6%) | 406  (71%) | 39  (6.8%) | 0.613 | 0.434 | | | 121 (21%) | 9  (1.6%) | 421  (73%) | 24  (4.2%) | 0.198 | 0.656 | 73.54 ± 33.35 | 51.69 ± 37.03 | 5.972 | **<0.001*** |
| Non-Rubiaceae data set | 65 (0.5%) | 48 (0.4%) | 8179 (65%) | 4399 (35%) | 2.451 | 0.118 | | | 93 (0.7%) | 20 (0.2%) | 11048 (87%) | 1530 (12%) | 2.705 | 0.100 | 81.24 ± 27.08 | 61.41 ± 34.99 | 6.115 | **<0.001*** |
|  |  |  |  |  |  |  | | |  |  |  |  |  |  |  |  |  |  |
| **(B) ALL GENERA OF OUR FINAL PHYLOGENY (9,425 GENERA)** | | | | |  |  | | |  |  |  |  |  |  |  |  |  |  |
|  | Tropical distribution | | | | | | | | Biodiversity hotspots | | | | | | Number of species of the genera | | | |
|  | Poly-TR | Poly-Non-TR | Non-poly-TR | Non-poly-Non-TR | X^2^ | p-value | | | Poly-HS | Poly-Non-HS | Non-poly-HS | Non-poly-Non-HS | X^2^ | p-value | Number of species of polymorphic genera (mean ± SD) | Number of species of non-polymorphic genera (mean ± SD) | t-student | p-value |
| Full data set using a 50% threshold for tropical distribution | 146 (1.5%) | 60 (0.6%) | 5805 (62%) | 3414 (36%) | 5.077 | **0.024*** | | | 180 (1.9%) | 26 (0.3%) | 8080 (86%) | 1139 (12%) | 0.000 | 0.994 | 81.61 ± 27.67 | 67.62 ± 33.13 | 6.010 | **<0.001*** |
| Full data set using a 70% threshold for tropical distribution | 132 (1.4%) | 74 (0.8%) | 4840 (51%) | 4379 (47%) | 10.377 | **0.001*** | | | / | / | / | / | / | / | / | / | / | / |
| Rubiaceae data set | 91 (24%) | 13 (3.4%) | 254  (66%) | 30  (7.7%) | 0.127 | 0.772 | | | 98 (25%) | 6  (1.6%) | 248  (64%) | 36  (9.3%) | 3.081 | 0.079 | 79.18 ± 30.26 | 61.51 ± 35.83 | 4.476 | **<0.001*** |
| Non-Rubiaceae data set | 55 (0.6%) | 47 (0.5%) | 5551 (61%) | 3384 (38%) | 2.545 | 0.111 | | | 82 (0.9%) | 20 (0.2%) | 7883 (87%) | 1052 (12%) | 5.194 | **0.023*** | 84.08 ± 24.66 | 67.82 ± 33.02 | 4.958 | **<0.001*** |

**Table S4.** Strength of phylogenetic signal (phylo D) of style-length polymorphism, tropical distribution using a 50% threshold, tropical distribution using a 70% threshold, and occurrence in biodiversity hotspots for three data sets: (A) full data set, (B) Rubiaceae data set, and (C) non-Rubiaceae data set. The observed D statistic and its simulation-based p-values comparing the observed pattern to two null expectations: a random distribution across the phylogeny (expected D ~ 1) and Brownian motion evolution (expected D ~ 0) are reported for each variable separately. Values of D < 0 indicate stronger phylogenetic conservatism than Brownian, values near 0 are consistent with Brownian motion, values near 1 indicate randomness, and values > 1 suggest overdispersion. * denotes cases where the observed D is not significantly different from Brownian expectations (p > 0.05). N state 0 = number of non-polymorphic genera/non-tropical/outside hotspot; N state 1 = number of polymorphic genera/tropical/within hotspot.

|  | phylo D | p resulting from no (random) phylogenetic structure | p resulting from Brownian phylogenetic structure | N state 0 | N state 1 |
| --- | --- | --- | --- | --- | --- |
| **(A) FULL DATA SET** |  |  |  |  |  |
| Style-length polymorphism | -0.026* | 0 | 0.612 | 9219 | 206 |
| Tropical distribution using a 50% threshold | 0.200 | 0 | 0 | 3474 | 5951 |
| Tropical distribution using a 70% threshold | 0.243 | 0 | 0 | 4453 | 4972 |
| Biodiversity hotspots | 0.596 | 0 | 0 | 1165 | 8260 |
| **(B) RUBIACEAE DATA SET** |  |  |  |  |  |
| Style-length polymorphism | 0.209* | 0 | 0.173 | 284 | 104 |
| Tropical distribution using a 50% threshold | 0.197* | 0 | 0.233 | 43 | 345 |
| Tropical distribution using a 70% threshold | 0.368* | 0 | 0.054 | 58 | 330 |
| Biodiversity hotspots | 1.009 | 0.516 | 0 | 45 | 343 |
| **(C) NON-RUBIACEAE DATA SET** |  |  |  |  |  |
| Style-length polymorphism | 0.010* | 0 | 0.518 | 8935 | 102 |
| Tropical distribution using a 50% threshold | 0.198 | 0 | 0 | 3431 | 5606 |
| Tropical distribution using a 70% threshold | 0.242 | 0 | 0 | 4395 | 4642 |
| Biodiversity hotspots | 1.010 | 0.772 | 0 | 1075 | 7962 |

**Table S5.** Statistical results of the phylogenetic logistic regression modeling style-length polymorphism as a function of tropical distribution (TR), presence in biodiversity hotspots (HS), number of species (NS), and phylogeny (PHY). Models were run including all predictors simultaneously, excluding the hotspot variable, and excluding the tropical distribution variable, for four data sets: (A) full data set using a 50% threshold for tropical distribution, (B) full data set using a 70% threshold for tropical distribution, (C) Rubiaceae data set, and (D) non-Rubiaceae data set.

1. **FULL DATA SET USING A 50% THRESHOLD FOR TROPICAL DISTRIBUTION**

| Model | Estimate | AIC | ΔAIC | AIC Weights |
| --- | --- | --- | --- | --- |
| **TR+HS+NS+PHY** | | 1084.133 | 3.945 | 0.104 |
| Intercept | -5.018 |  |  |  |
| TR | 0.430 |  |  |  |
| HS | -0.253 |  |  |  |
| NS | 0.010 |  |  |  |
| **TR+NS+PHY** |  | 1083.406 | 3.217 | 0.150 |
| Intercept | -5.188 |  |  |  |
| TR | 0.351 |  |  |  |
| NS | 0.010 |  |  |  |
| **HS+NS+PHY** |  | 1080.189 | 0.000 | 0.747 |
| Intercept | -4.968 |  |  |  |
| HS | 0.014 |  |  |  |
| NS | 0.010 |  |  |  |

1. **FULL DATA SET USING A 70% THRESHOLD FOR TROPICAL DISTRIBUTION**

| Model | Estimate | AIC | ΔAIC | AIC Weights |
| --- | --- | --- | --- | --- |
| **TR+HS+NS+PHY** | | 1088.279 | 8.091 | 0.017 |
| Intercept | -5.050 |  |  |  |
| TR | 0.557 |  |  |  |
| HS | -0.274 |  |  |  |
| NS | 0.010 |  |  |  |
| **TR+NS+PHY** |  | 1090.279 | 9.848 | 0.007 |
| Intercept | -5.250 |  |  |  |
| TR | 0.491 |  |  |  |
| NS | 0.010 |  |  |  |
| **HS+NS+PHY** |  | 1080.189 | 0.000 | 0.976 |
| Intercept | -4.968 |  |  |  |
| HS | 0.014 |  |  |  |
| NS | 0.010 |  |  |  |

1. **RUBIACEAE DATA SET**

| Model | Estimate | AIC | ΔAIC | AIC Weights |
| --- | --- | --- | --- | --- |
| **TR+HS+NS+PHY** | | 346.735 | 6.005 | 0.042 |
| Intercept | -2.291 |  |  |  |
| TR | -0.280 |  |  |  |
| HS | 0.446 |  |  |  |
| NS | 0.016 |  |  |  |
| **TR+NS+PHY** |  | 340.730 | 0.000 | 0.835 |
| Intercept | -1.885 |  |  |  |
| TR | -0.284 |  |  |  |
| NS | 0.013 |  |  |  |
| **HS+NS+PHY** |  | 344.560 | 3.830 | 0.123 |
| Intercept | -2.531 |  |  |  |
| HS | 0.449 |  |  |  |
| NS | 0.015 |  |  |  |

1. **NON-RUBIACEAE DATA SET**

| Model | Estimate | AIC | ΔAIC | AIC Weights |
| --- | --- | --- | --- | --- |
| **TR+HS+NS+PHY** | | 722.215 | 4.633 | 0.090 |
| Intercept | -5.095 |  |  |  |
| TR | -0.346 |  |  |  |
| HS | -0.761 |  |  |  |
| NS | 0.016 |  |  |  |
| **TR+NS+PHY** |  | 730.936 | 13.354 | 0.001 |
| Intercept | -5.738 |  |  |  |
| TR | -0.348 |  |  |  |
| NS | 0.016 |  |  |  |
| **HS+NS+PHY** |  | 717.583 | 0.000 | 0.909 |
| Intercept | -5.291 |  |  |  |
| HS | -0.762 |  |  |  |
| NS | 0.016 |  |  |  |

**Table S6.** Partial contributions of tropical distribution (TR), occurrence in biodiversity hotspots (HS), number of species (NS), and phylogeny (PHY) to the occurrence of style-length polymorphism using four data sets: (A) full data set using a 50% threshold for tropical distribution, (B) full data set using a 70% threshold for tropical distribution, (C) Rubiaceae data set, and (D) non-Rubiaceae data set. Three model configurations were considered as the full model set: including all predictors simultaneously, excluding the hotspot variable, and excluding the tropical distribution variable. The R^2^_lik_ corresponds to the variable excluded from the model.

| **(A) FULL DATA SET USING A 50%**  **THRESHOLD FOR TROPICAL DISTRIBUTION** |  |  |  |  |
| --- | --- | --- | --- | --- |
| Full model | Reduced model | R_2_lik(%) | ΔlogLik (full-reduced) | p-value |
| TR+HS+NS+PHY | 1 | 48.4 | 908.508 | <0.001* |
| TR+HS+NS+PHY | TR+HS+NS | 47.1 | 861.942 | <0.001* |
| TR+HS+NS+PHY | TR+HS+PHY | 1.8 | 18.868 | <0.001* |
| TR+HS+NS+PHY | TR+NS+PHY | 1.2 | 1.272 | 0.259 |
| TR+HS+NS+PHY | HS+NS+PHY | 0.0 | -1.944 | 1.000 |
| TR+NS+PHY | 1 | 48.5 | 907.236 | <0.001* |
| TR+NS+PHY | TR+NS | 47.0 | 861.743 | <0.001* |
| TR+NS+PHY | TR+PHY | 1.9 | 19.593 | <0.001* |
| TR+NS+PHY | NS+PHY | 0.0 | -3.275 | 1.000 |
| HS+NS+PHY | 1 | 48.4 | 910.452 | <0.001* |
| HS+NS+PHY | HS+NS | 47.3 | 870.304 | <0.001* |
| HS+NS+PHY | HS+PHY | 2.3 | 23.917 | <0.001* |
| HS+NS+PHY | NS+PHY | 0.0 | -0.058 | 1.000 |
| **(B) FULL DATA SET USING A 70% THRESHOLD FOR TROPICAL DISTRIBUTION** | | | |  |
| Full model | Reduced model | R_2_lik(%) | ΔlogLik (full-reduced) | p-value |
| TR+HS+NS+PHY | 1 | 48.2 | 900.604 | <0.001* |
| TR+HS+NS+PHY | TR+HS+NS | 46.6 | 851.268 | <0.001* |
| TR+HS+NS+PHY | TR+HS+PHY | 1.5 | 15.619 | <0.001* |
| TR+HS+NS+PHY | TR+NS+PHY | 3.6 | 3.757 | 0.052 |
| TR+HS+NS+PHY | HS+NS+PHY | 0.0 | -6.091 | 1.000 |
| TR+NS+PHY | 1 | 48.5 | 900.605 | <0.001* |
| TR+NS+PHY | TR+NS | 46.5 | 848.876 | <0.001* |
| TR+NS+PHY | TR+PHY | 1.5 | 15.874 | <0.001* |
| TR+NS+PHY | NS+PHY | 0.0 | -9.906 | 1.000 |
| HS+NS+PHY | 1 | 48.0 | 910.452 | <0.001* |
| HS+NS+PHY | HS+NS | 47.4 | 870.304 | <0.001* |
| HS+NS+PHY | HS+PHY | 2.3 | 23.917 | <0.001* |
| HS+NS+PHY | NS+PHY | 0 | -0.058 | 1.000 |
| **(C) RUBIACEAE DATA SET** |  |  |  |  |

| Full model | Reduced model | R_2_lik(%) | ΔlogLik (full-reduced) | p-value |
| --- | --- | --- | --- | --- |
| TR+HS+NS+PHY | 1 | 42.6 | 134.451 | <0.001* |
| TR+HS+NS+PHY | TR+HS+NS | 37.5 | 112.225 | <0.001* |
| TR+HS+NS+PHY | TR+HS+PHY | 0.4 | 1.019 | 0.313 |
| TR+HS+NS+PHY | TR+NS+PHY | 0.0 | -7.699 | 1.000 |
| TR+HS+NS+PHY | HS+NS+PHY | 0.0 | -0.390 | 1.000 |
| TR+NS+PHY | 1 | 37.2 | 118.398 | <0.001* |
| TR+NS+PHY | TR+NS | 36.2 | 97.454 | <0.001* |
| TR+NS+PHY | TR+PHY | 0.3 | -13.560 | 1.000 |
| TR+NS+PHY | NS+PHY | 0.0 | 0.457 | 0.499 |
| HS+NS+PHY | 1 | 38.2 | 114.530 | <0.001* |
| HS+NS+PHY | HS+NS | 35.8 | 92.875 | <0.001* |
| HS+NS+PHY | HS+PHY | 0.7 | -18.619 | 1.000 |
| HS+NS+PHY | NS+PHY | 1.5 | -3.373 | 1.000 |
| **(D) NON-RUBIACEAE DATA SET** | | | |  |
| Full model | Reduced model | R_2_lik(%) | ΔlogLik (full-reduced) | p-value |
| TR+HS+NS+PHY | 1 | 37.7 | 405.388 | <0.001* |
| TR+HS+NS+PHY | TR+HS+NS | 35.2 | 365.438 | <0.001* |
| TR+HS+NS+PHY | TR+HS+PHY | 3.5 | 24.863 | <0.001* |
| TR+HS+NS+PHY | TR+NS+PHY | 1.5 | 10.721 | 0.001* |
| TR+HS+NS+PHY | HS+NS+PHY | 0.0 | -2.633 | 1.000 |
| TR+NS+PHY | 1 | 37.9 | 394.667 | <0.001* |
| TR+NS+PHY | TR+NS | 34.8 | 363.448 | <0.001* |
| TR+NS+PHY | TR+PHY | 3.3 | 23.582 | <0.001* |
| TR+NS+PHY | NS+PHY | 0.0 | -1.879 | 1.000 |
| HS+NS+PHY | 1 | 36.7 | 408.020 | <0.001* |
| HS+NS+PHY | HS+NS | 35.6 | 371.015 | <0.001* |
| HS+NS+PHY | HS+PHY | 3.8 | 27.185 | <0.001* |
| HS+NS+PHY | NS+PHY | 1.7 | 11.474 | <0.001* |
|  | | | |  |

**Table S7.** Moran’s autocorrelation Index for the three data sets: full data set using a 50% threshold for tropical distribution (N non-polymorphic = 9219; N polymorphic = 206), Rubiaceae data set (N non-polymorphic = 284; N polymorphic = 104), and (C) non-Rubiaceae data set (N non-polymorphic = 8935; N polymorphic = 102).

|  | | | | |
| --- | --- | --- | --- | --- |
|  | observed | expected | sd | p-value |
| Full data set using a 50% threshold for tropical distribution | 0.001 | -0.000 | 0.001 | 0.173 |
| Rubiaceae data set | 0.008 | -0.003 | 0.008 | 0.219 |
| Non-Rubiaceae data set | 0.002 | -0.000 | 0.001 | 0.001* |
|  |  |  |  |  |
